# Syllabus of Eclectic Materia Medica and Therapeutics

**Published:** 1895-11

**Authors:** 


Syllabus of Eclectic Materia Medica and Therapeutics. Compiled from notes taken from the lectures of Frederick J. Locke, M. D., Dean of Faculty and Professor of Materia Medica and Therapeutics in the Eclectic Medical Institute, Cincinnati, Ohio. Edited with Pharmacological additions by Harvey W. Felter, M. D. With notes on specific medicines by John Uri Lloyd. Cincinnati, Ohio: John M. Scudder & Sons. 1895. Cloth. 8vo. Price, $2.50 net.

The motive for the publication of this book may be found in the preface. "The urgent demands, repeated from year to year, by the students and graduates of the Eclectic Medical Institute who have listened to the lectures on materia medica in that institution, that Professor Locke should prepare a work on this subject." The book consists really of a series of notes on well-known drugs.

The classification of the drugs is into the same general groupings that distinguish the materia medica of the old school of medicine. There are emetics, cathartics, diaphoretics, diuretics, sedatives, narcotics, stimulants, and all the rest of the well-known divisions. Each drug is treated with regard to its botanical origin, its chief active constituent, the essential qualities of the specific variety used in medicine, and its therapeutic uses upon the lines of the above-stated classification. The diseases in which experience proves the drug to have been useful are also given with great particularity and yet commendable brevity. Of course there are no indications for the remedy from the homœopathic standpoint, and therefore the book will hardly be of very much use to homœopathic physicians. Yet there are lots of points of information which even the homœopathist may desire, to be found in its pages. It is specially commendable by reason of its brevity, compactness, and clearness. It has two indexes, a clinical index and a subject index, both alphabetically arranged, which greatly enhance its value. Among physicians practicing the eclectic method, it must become very popular, and to students in the Eclectic Colleges it is indispensable.

